# A novel mechanism of auxin habituation: upregulation of auxin receptor TRANSPORT INHIBITOR RESPONSE 1 allows cell proliferation independent of external auxin

**DOI:** 10.1111/nph.70763

**Published:** 2025-12-09

**Authors:** Pavel Jelínek, Karel Müller, Eliška Kobercová, Adéla Přibylová, Milada Čovanová, Petre I. Dobrev, Roberta Vaculíková, Zuzana Vondráková, Lenka Helusová, Anita Bírošíková, Lukáš Fischer, Jan Petrášek

**Affiliations:** ^1^ Department of Experimental Plant Biology, Faculty of Science Charles University Viničná 5 128 00 Prague 2 Czech Republic; ^2^ Institute of Experimental Botany of the Czech Academy of Sciences Rozvojová 263 165 00 Prague 6 Czech Republic

**Keywords:** auxin, auxin autonomy, cell division, cell line, habituation, *Nicotiana tabacum*, TIR1

## Abstract

- Exogenously applied auxins are essential for establishing cell lines in tissue cultures and maintaining their proliferation. Cell lines may develop the ability to proliferate even in media lacking auxin, they may become auxin‐habituated. This study investigated the mechanisms underlying this process.
- Here, we conducted comprehensive auxin metabolic profilings, pharmacological treatments and transcriptomic comparisons in two independently habituated tobacco cell lines, BY‐2H and VBI‐2, derived from cell lines of cultivars Bright Yellow (BY‐2) and Virginia Bright Italia (VBI‐0).
- Our results show that both habituated lines developed different mechanisms of auxin autonomy. In VBI‐2, increased expression of MADS‐domain transcription factor genes suggests epigenetically determined habituation. By contrast, in BY‐2H, genome resequencing identified a massive amplification of the genomic region containing the TRANSPORT INHIBITOR RESPONSE 1 (TIR1) gene, causing its strong upregulation. Mimicking this by inducible overexpression of TIR1 in the auxin‐dependent BY‐2 line allowed its proliferation in the absence of exogenous auxin.
- Compensating for auxin deficiency by increasing level of its receptor is a very intriguing phenomenon. The amplification of the TIR1 genomic region is a unique example of in‐flask microevolution under strong selection pressure with potential interest for biotechnological applications.

## Shrnutí

- Exogenně aplikovaný rostlinný hormon auxin je nezbytný pro založení buněčných linií v tkáňových kulturách a pro udržení jejich proliferace. Některé buněčné linie však mohou získat schopnost růst i v médiu bez přídavku auxinu – stávají se tzv. habituované. Tato studie se zabývala mechanismy, které tento proces navozují.
- U dvou nezávisle habituovaných tabákových buněčných linií, BY‐2H a VBI‐2, odvozených od auxin‐závislých linií Bright Yellow (BY‐2) a Virginia Bright Italia (VBI‐0), jsme provedli komplexní profilování metabolismu auxinu, farmakologické testy inhibitorové odezvy a transkriptomické porovnání.
- Naše výsledky ukazují, že obě habituované linie vyvinuly odlišný mechanismus auxinové autonomie. Zatímco v případě VBI‐2 linie, kde zvýšená exprese genů transkripčních faktorů rodiny MADS naznačuje epigeneticky determinovanou dráhu, v linii BY‐2H bylo identifikováno masivní pomnožení genomové oblasti obsahující gen kodující receptor auxinu TRANSPORT INHIBITOR RESPONSE1 (TIR1), které vedlo k jeho výrazné nadregulaci. Napodobením tohoto jevu expresí indukovatelným TIR1 v auxin‐závislé linii BY‐2 bylo dosaženo schopnosti jejího dělení bez přítomnosti vnějšího auxinu.
- Kompenzace nedostatku auxinu zvýšením hladiny jeho receptoru je velmi zajímavým jevem. Amplifikace genomové oblasti TIR1 je jedinečným příkladem mikroevoluce v laboratorních podmínkách pod silným selekčním tlakem, a může mít významný potenciál pro biotechnologické aplikace.

## Introduction

Indole‐3‐acetic acid (IAA) is the most abundant and best characterized naturally occurring auxin. Other compounds with auxin activity have been identified, including 4‐chloro‐indole‐3‐acetic acid, phenylacetic acid, as well as synthetic 2,4‐dichlorophenoxyacetic acid (2,4‐D) and 1‐naphthylacetic acid (NAA; Simon & Petrášek, 2011). Auxins exert their effects through a number of signaling pathways. Based on studies in *Arabidopsis thaliana*, in the canonical signaling pathway, IAA binds to auxin receptors TRANSPORT INHIBITOR RESPONSE 1 (TIR1) and AUXIN F‐BOX 2, 3, 4, 5 (AFB2, 3, 4, 5). IAA binding allows for the interaction between the auxin and the AUXIN/INDOLE‐3‐ACETIC ACID (AUX/IAA) transcriptional repressors, which undergo ubiquitination and degradation upon interaction with TIR1/AFBs (Dharmasiri *et al*., 2005a,b). Following the degradation of the repressor, type A AUXIN RESPONSE FACTORs (ARFs) released from the interaction with AUX/IAA promote the expression of auxin‐induced genes (Leyser, 2018; Hernández‐García *et al*., 2024; Jaiswal *et al*., 2024). An additional mode of action has been proposed based on the adenylate cyclase activity of TIR1 and AFB5 (Qi *et al*., 2022), or the guanylyl cyclase activity of AFB1 (Qi *et al*., 2023). Moreover, signaling by cytoplasmic AFB1 in rapid nontranscriptional inhibition of root growth was shown to include membrane depolarization and calcium efflux (Serre *et al*., 2021; Chen *et al*., 2023; Dubey *et al*., 2023). Finally, the signaling pathway dependent on the extracellular perception of auxin by AUXIN BINDING PROTEIN 1 interacting with TRANSMEMBRANE KINASE initiates a specific phosphorylation signal within the cell (Friml *et al*., 2022; Yu *et al*., 2023; Rodriguez et al., 2025).

The regulation of auxin homeostasis occurs at multiple levels, with the most extensively studied aspect being the transport of auxin. This encompasses both free transport across the cell membrane by diffusion and transport facilitated by proteins (Hammes & Pedersen, 2024). In contrast to the field of auxin transport, the area of auxin metabolism remains relatively understudied. Based on the identified precursors, several auxin biosynthetic pathways have been postulated; however, only one pathway has been fully characterized, including the precursor indole‐3‐pyruvate and activities of enzymes from the TRYPTOPHAN AMINO TRANSFERASE (TAA) and flavin‐containing monooxygenases (YUCCA) gene families (Mashiguchi *et al*., 2011; Cao *et al*., 2019). Furthermore, IAA is metabolized by a series of enzymatic reactions that contribute to the fine‐tuning of free IAA levels. IAA can be reversibly inactivated by conjugation with amino acids and sugars, or irreversibly inactivated by oxidation catalyzed by DIOXYGENASE FOR AUXIN OXIDATION1 (LeClere *et al*., 2002; Staswick *et al*., 2005; Casanova‐Sáez *et al*., 2021; Hayashi *et al*., 2021; Müller *et al*., 2021). In general, the interplay between auxin transport, metabolism and signaling controls plant growth and development (Vanneste *et al*., 2025).

The evolution of plants has led to remarkable developmental adaptability, achieved through tightly controlled cell divisions and differentiation (Bowles *et al*., 2023). Plant cell lines, now also listed and annotated in Cellosaurus (Bairoch, 2018), represent a simplified system, in which regulated cell division and differentiation are achieved under specific conditions (Maruyama *et al*., 2020; Bapat *et al*., 2023). While the main progress in the cultivation and establishment of plant cell lines was made in the middle of the last century (Heller, 1953; Murashige & Skoog, 1962; Thorpe, 2007), their applications in plant cell‐based bioproduction are still limited (Xu *et al*., 2025).

For the optimal growth of the majority of cell lines, the presence of exogenously applied synthetic auxin like 2,4‐D is required (Matsumoto *et al*., 1971; Opatrný & Opatrná, 1976; Yokoya & Handro, 1996; Ngomuo *et al*., 2013; Mayerni *et al*., 2020). A reduced concentration or withdrawal of external auxin typically arrests the cell cycle in G1 or G2 phases. The application of auxin on the other hand stimulates the progression through G1/S and G2/M checkpoints by the induction of specific CYCLINs and CYCLIN‐DEPENDENT KINASEs (Shimotohno *et al*., 2021).

In plant research, one of the most significant cell lines is the tobacco cell line BY‐2, established in the late 60s from *Nicotiana tabacum* L. cv Bright Yellow 2 plants and primarily used in the tobacco industry at the Central Research Institute of Japan Monopoly Corp. It was later established as a cellular model for basic research of the cell cycle, membrane and cytoskeletal dynamics, and many others (Matsumoto *et al*., 1971; Kato *et al*., 1972; Nagata *et al*., 1992, 2004). By the process of habituation, at least two cell lines independent of exogenous auxin addition were derived from the original BY‐2 cell line. The 2B‐13 line was habituated by decreasing auxin concentration in the media and UV irradiation (Shimizu *et al*., 2006); however, it has been lost without understanding of the habituation mechanism (Nagata et al., 2023). Fortunately, another auxin‐habituated line BY‐2H, derived by cultivation of cells in the media with low IAA concentrations (Syono & Furuya, 1974) can be obtained from the Riken database (Syono & Fujita, 1994). In addition, auxin habituation was also induced in the tobacco cell line derived from the cultivar Virginia Bright Italia (Opatrný & Opatrná, 1976; Qiao *et al*., 2010).

In this work, we utilized pairs of auxin‐dependent and auxin‐habituated cell lines, that is BY‐2/BY‐2H and VBI‐0/VBI‐2 (established in this work). We present a thorough metabolomic, pharmacological and transcriptomic characterization of these lines and show that the mechanism of habituation is different in the two habituated lines. While we hypothesize about epigenetic causes in VBI‐2 cells, in the BY‐2H line we succeeded in discovering a novel mechanism of auxin habituation based on the tobacco *TIR1* homolog multiplication, which confers sensitivity to very low levels of auxin in this line.

## Materials and Methods

### Plant material and chemicals

BY‐2 cell line (*Nicotiana tabacum* L. cv Bright Yellow 2) was cultured in Murashige and Skoog (MS) medium (3% sucrose (w/v), 4.3 g l^−1^ MS salts, 100 mg l^−1^ myo‐inositol, 1 mg l^−1^ thiamin, 0.2 mg l^−1^ 2,4‐D and 200 mg l^−1^ KH_2_PO_4_; pH 5.8; Murashige & Skoog, 1962; Kato *et al*., 1972). Habituated BY‐2H cell line rpc00036 was obtained from the Riken BRC experimental plant web and cultured in MS medium without 2,4‐D. VBI‐0 cell line (*Nicotiana tabacum* cv. Virginia Bright Italia) was cultured in modified Heller medium supplemented with 1 mg l^−1^ 2,4‐D and 1 mg l^−1^ NAA (Heller, 1953; Opatrný & Opatrná, 1976). Habituated VBI‐2 cell line, derived from the original VBI‐0 by the protocol described in Qiao *et al*. (2010), was cultured in modified Heller medium without the supplemented auxins. Suspension cultures were maintained under continuous shaking (150 rpm; orbital diameter 30 mm), and subcultured every 7 (BY‐2) and 14 (BY‐2H, VBI‐0 and VBI‐2) days. Stock calli were maintained on the same media solidified with 0.7% (w/v) agar and subcultured monthly. All cultivations were performed in the dark at 27°C. All chemicals were purchased from Merck unless stated otherwise.

### Treatments

Inhibitors of auxin biosynthesis Yucasine DF obtained from Prof. Ken‐Ichiro Hayashi (Okayama University, Japan) and l‐kynurenine, both dissolved in dimethyl sulfoxide (DMSO; He *et al*., 2011; Tsugafune *et al*., 2017), were added in final concentrations of 20 and 2 μM, respectively, at the beginning of the subculture interval in combined treatments. Effects on cell division and auxin metabolism were scored after two (BY‐2) or three (BY‐2H, VBI‐0, VBI‐2) days in cell suspensions and after 2 wk in calli (He *et al*., 2011; Tsugafune *et al*., 2017). The effects of inhibitors of auxin signaling auxinole and PEO‐IAA (both dissolved in DMSO; Hayashi *et al*., 2008, 2012) were scored after 2 d in suspensions. The final concentrations of the inhibitor were 5, 10, 20, 50 and 100 μM. The same amount of the solvent was added to the control samples. Cell densities were determined by counting cells in at least 10 aliquots of each sample using a Fuchs‐Rosenthal hemocytometer slide.

### Gene construct preparation and transformation of cells

All vectors for transformation were prepared using the GoldenBraid 2.0 cloning system (Sarrion‐Perdigones *et al*., 2013). The DNA parts of the system were obtained from Diego Orzaez (Addgene Kit, no.: 1000000076) or the MoClo Toolkit, from Sylvestre Marillonnet (Addgene Kit, no.: 1000000044). For inducible upregulation of transcription, GoldenBraid‐compatible components from the β‐estradiol‐inducible expression system (Zuo *et al*., 2000) were used. The specific cDNA parts were prepared in the Laboratory of Hormonal Regulation in Plants IEB, Prague. The BsmBI and BsaI (Thermo Fisher Scientific, Waltham, MA, USA, or New England Biolabs, Ipswich, MA, USA) type II restriction cloning enzymes were used. The T4 ligase (Promega) was used for the ligation of individual cloning parts. For upregulation of genes involved in auxin biosynthesis, *NtTAA1T* and *NtYUCCA3T* coding sequences (GenBank IDs XM_016652894.1 and XM_016640518.1, respectively) were cloned. For inducible upregulation of auxin perception, a coding sequence of *NtTIR1T* (GenBank ID XM_016613973.1) was used. The transformation of 3‐d‐old tobacco cells (Petrášek *et al*., 2003) was performed by co‐incubation with *Agrobacterium tumefaciens* strain GV2260 (An *et al*., 1985). Transformants were regenerated on selection media containing 20 μg ml^−1^ Hygromycin B and 100 μg ml^−1^ Cefotaxime. For the induction of expression of gene constructs carrying the XVE system, 1 μM β‐estradiol was used. The positive transformants were screened using reverse transcription‐quantitative polymerase chain reaction (RT‐qPCR) or by light microscopy. Two lines with the most profound upregulation of the target were used for functional experiments.

### RNA isolation and sequencing analysis

Total RNA was isolated from 50 to 100 mg of cells using the RNeasy Plant Mini Kit (Qiagen) or Spectrum™ Plant Total RNA Kit (Sigma‐Aldrich) and treated with the DNA‐Free Kit (Thermo Fisher Scientific). RNA purity, concentration and integrity were evaluated on 0.8% agarose gels (v/w) and by the RNA Nano 6000 Assay Kit using the Bioanalyzer instrument (Agilent Technologies). For RNA‐seq analysis, *c*. 5 μg of RNA was submitted for the service procedure provided by GATC‐Biotech or Novogene companies. The analysis resulted in at least 15 million 50‐bp long reads (BY‐2, VBI‐0 and VBI‐2 samples) or at least 15 million 150‐bp read pairs (BY‐2H samples). Rough reads were quality‐filtered using Rcorrector and Trim Galore scripts (Song & Florea, 2015). Transcript abundances (transcripts per million – TPM) were determined using Salmon (Patro *et al*., 2017) with parameters ‐‐posBias, ‐‐seqBias, ‐‐gcBias, ‐‐numBootstraps 30. An index was built from the *Nicotiana tabacum* v.1.0 cDNA dataset (Edwards *et al*., 2017). Visualization, quality control of data analysis and determination of differentially expressed genes (DEGs) were conducted using the sleuth (v.0.29.0) package in R (Pimentel *et al*., 2017). Transcripts with *q*‐value ≤ 0.05 and log_2_ fold change ≥ 1 (upregulated) or ≤ −1 (downregulated) were considered to be significantly differentially expressed. Other criteria for the selection of the gene list of interest are specified in the corresponding results section. Gene Ontology (GO) analysis (statistical overrepresentation test) was performed using the Panther Classification System (Mi *et al*., 2019).

### DNA isolation and sequencing

Genomic DNA was isolated from *c*. 50 mg of freshly collected BY‐2 and BY‐2H cells using the DNeasy Plant Mini Kit. Two milligrams of purified DNA was submitted for whole‐genome resequencing service provided by the Institute of Applied Biotechnologies (IAB) Co. A total of 792 and 652 million read pairs (150 bps each) were obtained for the BY‐2 and BY‐2H lines, respectively. Rough reads were processed using the fastp tool (Chen, 2023) and mapped against the *Nicotiana tabacum* reference genome (Nitab v.1.0 Scf Edwards 2017; Edwards *et al*., 2017) using Bwa (Li & Durbin, 2010). Resulting BAM files were sorted, and duplicated reads were removed using Picard tools (https://broadinstitute.github.io/picard/). Coverage depth was assessed by samtools (Danecek *et al*., 2021).

### Quantitative polymerase chain reaction

Gene copy numbers of *NtTIR1T* and *NtTIR1S* were estimated using quantitative real‐time PCR. The reaction was performed using GoTaq qPCR Master Mix (Promega) at 58°C annealing temperature on a LightCycler480 instrument (Roche). 20× diluted DNA isolated as described above was used as the reaction template. Sequences of specific primers are listed in Supporting Information Table S1. The ratio of *NtTIR1T*/*NtTIR1S* gene copies was calculated using the equation:

$$\text{ratio}=\frac{{\mathrm{eff}}_{\text{NtTIR}1S}^{{\mathrm{CP}}_{\text{NtTIR}1S}}}{{\mathrm{eff}}_{\text{NtTIR}1T}^{{\mathrm{CP}}_{\text{NtTIR}1T}}}$$

where eff stands for qPCR efficiency estimated using a serial dilution of template DNA, and CP stands for the respective crossing points. The experiment was done using three independently isolated DNAs and two technical repetitions of qPCR reaction.

### Reverse transcription ‐ quantitative polymerase chain reaction

Approximately 1 μg of DNAse‐treated RNA was reverse‐transcribed using M‐MLV reverse transcriptase, RNase H‐, point mutant (Promega). RT‐qPCR was performed using GoTaq qPCR Master Mix (Promega) at 58°C annealing temperature on a LightCycler480 instrument (Roche). PCR efficiency was estimated using serial dilution of template cDNA. Tobacco elongation factor 1a and aminopeptidase‐like genes (GenBank accession nos.: NM_001326165 and XM_016640300, respectively) were used as references for validating RNA‐seq data. Relative transcript abundancies were then calculated using the equation:

$$\mathrm{rel}.\text{transcript level}=\frac{\sqrt{{\mathrm{eff}}_{\mathrm{ref}1}^{{\mathrm{CP}}_{\mathrm{ref}1}}.{\mathrm{eff}}_{\mathrm{ref}2}^{{\mathrm{CP}}_{\mathrm{ref}2}}}}{{\mathrm{eff}}_{\text{target}}^{{\mathrm{CP}}_{\text{target}}}}$$

where eff_ref_ and eff_target_ stand for the qPCR efficiencies of reference and target genes, respectively, and CP_ref_ and CP_target_ stand for the respective crossing points of reference and targets genes. Positive transcript levels and the quality of PCR products were verified by melting curve analysis. Primer sequences are shown in Table S1.

### Gene annotations

Individual genes were annotated using the same principle as in Müller *et al*. (2021). In general, the annotation of genes followed information available at SOL Genomics Network under *Nicotiana tabacum* TN90 and *Nicotiana tabacum* v1.0 datasets (Sierro *et al*., 2014; Edwards *et al*., 2017). GenBank IDs were assigned according to the closest homolog using blastn. For enhanced clarity, selected genes were named according to the closest homolog in *Arabidopsis thaliana*. Endings of the name (*S* or *T*) correspond to the closest homolog in either *Nicotiana sylvestris* or *Nicotiana tomentosiformis*.

Selection of genes related to auxin homeostasis and signal transduction was based on the annotation of *Arabidopsis thaliana*, which included 522 genes assigned to the GO terms ‘response to auxin’ or ‘auxin‐activated signaling pathway’. In order to get a more complete image, we manually added genes related to auxin biosynthesis, transport and metabolism to the set. Using the tblastx function, we found their closest homologs in the *Nicotiana tabacum* reference dataset (994 transcripts of which 851 were distinguished as detected in at least one sample group). The resulting annotation of tobacco transcripts/genes is included in the supplementary data file (Table S4, to be described later).

Selection of strong candidate transcription factors (TFs) that might be important for the proliferation of the auxin‐habituated lines, we defined the following stringent criteria: transcript abundance (TPM) in all auxin‐dependent (BY‐2, VBI‐0) samples ≤ 0.5 and in all auxin‐habituated samples (BY‐2H or VBI‐2) ≥ 1 (Table S6, to be described later).

### IAA metabolic profiling

For the estimation of endogenous levels of IAA and its metabolites, 2‐d‐old BY‐2 and 5‐d‐old BY‐2H, VBI‐0 and VBI‐2 were cultured in their respective media without any additional supplements. For the purification of IAA metabolites, samples were collected (50–100 mg of cells per sample) by filtrating the cells from the media and immediately transferring them to liquid nitrogen. Extraction and purification were performed as described in Dobrev & Kamínek (2002) and Dobrev *et al*. (2017, 2023). For the IAA profiling experiments, IAA (1 μM) was added to the cells for 2 h, and then, the cells were collected as described previously. The analysis was performed by liquid chromatography mass spectrometry (LC‐MS) consisting of UHPLC 1290 Infinity II (Agilent Technologies) coupled to a 6495 triple‐quadrupole mass spectrometer (Agilent Technologies, Morges, Germany). For MS analysis, the multiple reaction monitoring mode with the isotope dilution method was used. Data acquisition and processing were done with the Mass Hunter software v.B.08 (Agilent Technologies).

### MS/MS protein analysis

Approximately 50 mg of fresh weight was harvested from exponential suspension cells (2‐d‐old BY‐2 and 3‐d‐old BY‐2H). Samples were frozen in liquid nitrogen and sent to the Laboratory of Mass Spectrometry at BIOCEV research centre, Faculty of Science, Charles University. For the following analysis, samples were lysed by boiling at 95°C for 10 min in 100 mM triethylammonium bicarbonate (TEAB) containing 2% sodium deoxycholate (SDC), 40 mM chloroacetamide, 10 mM Tris(2‐carboxyethyl)phosphine (TCEP) and further sonicated (Bandelin Sonoplus Mini 20, MS 1.5). Samples were further processed using SP3 beads on the Thermo KingFisher Flex automated Extraction & Purification System in a 96‐well plate. After washing, samples were digested in 50 mM TEAB at 40°C with 1 μg of trypsin for 2 h; then, another 1 μg of trypsin was added and digested overnight. After digestion, samples were acidified with trifluoracetic acid to 1% final concentration and peptides were desalted using in‐house made stage tips packed with C18 disks (Empore) according to Rappsilber *et al*. (2007). A nano reversed‐phase column (Ion Opticks Aurora Ultimate XT 25 cm × 75 μm ID, C18 UHPLC column, 1.7 μm particles, 120 Å pore size) was used for LC‐MS analysis. Eluting peptide cations were converted to gas‐phase ions by electrospray ionization and analyzed on a Thermo Orbitrap Ascend (Thermo Scientific). Tandem MS was performed by isolation at 1.5 Th with the quadrupole, higher‐energy collisional dissociation fragmentation with normalized collision energy of 30, and rapid scan MS analysis in the ion trap. Three biological replicates were analyzed.

## Results

### Endogenous levels of IAA and its metabolites supported by pharmacology treatments indicate auxin autonomy of BY‐2H and VBI‐2 auxin‐habituated cell lines

**Fig. 1:**
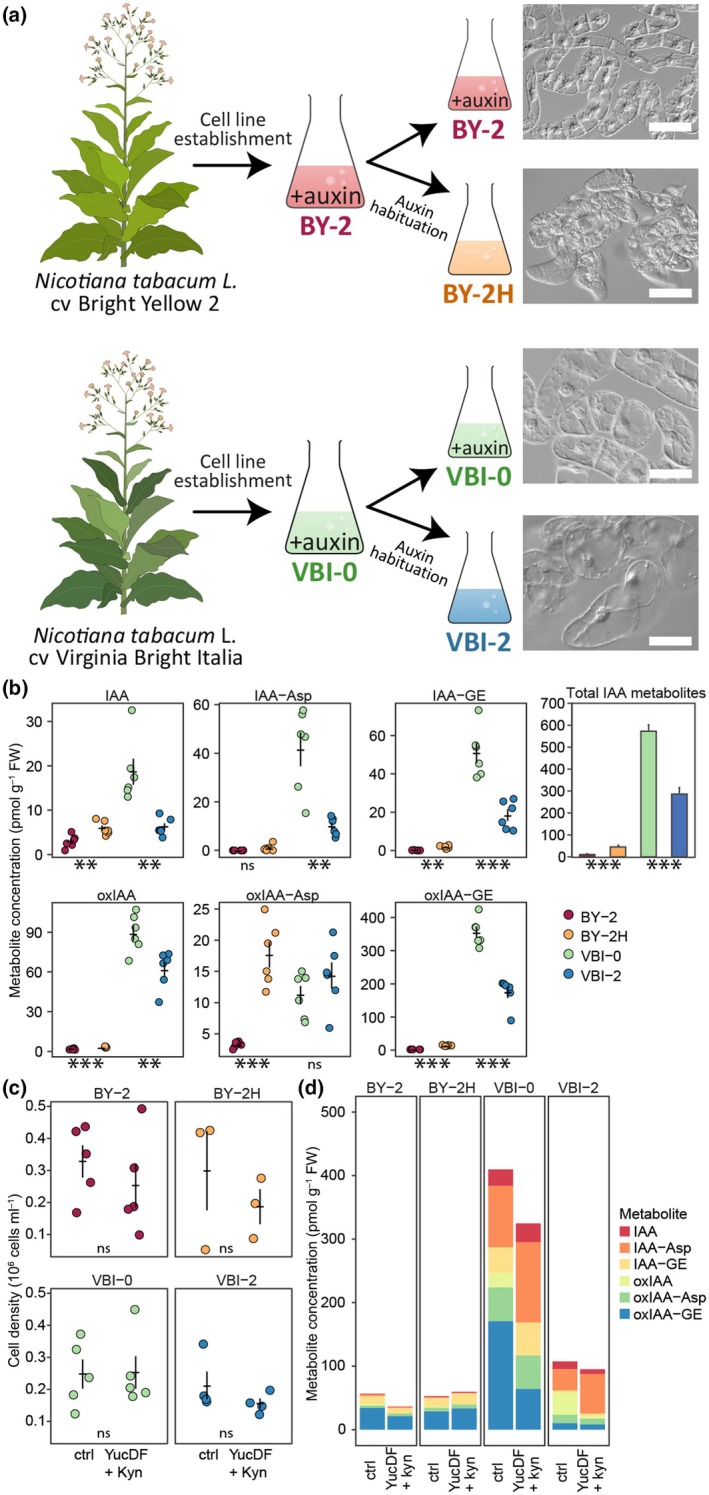
Endogenous levels of indole‐3‐acetic acid (IAA) in auxin‐habituated lines. (a) Scheme depicting the origin of tobacco cell lines used in this study, together with their cellular phenotypes. Bars, 100 μm. (b) Levels of endogenous free IAA and IAA metabolites in suspension cultures of tobacco cell lines in the exponential phase of growth. The significance of the difference in BY‐2H vs BY‐2 and VBI‐2 vs VBI‐0 comparisons was tested by Student's *t*‐test with the number of asterisks indicating *P*‐value, *P*‐values: **, *P* < 0.01; ***, *P* < 0.001; ns indicates not significant. Error bars indicate average metabolite concentration ±SE. The bar chart represents values of the sum of the concentration of IAA and all detected IAA metabolites. (c) Effects of Yucasin DF/l‐kynurenine (YucDF + Kyn) treatment (20 and 2 μM, respectively) on cell growth in suspension culture. Data points represent mean cell density in samples from at least five aliquots of at least three independent cell culture inoculations. Error bars indicate average cell density ± SE. (d) Effects of Yucasin DF/l‐kynurenine treatment on levels of IAA and its metabolites in calli. IAA‐Asp, indole‐3‐acetyl‐aspartate; IAA‐GE, indole‐3‐acetyl‐1‐glucosyl ester; oxIAA, 2‐oxindole‐3‐acetic acid; oxIAA‐Asp, 2‐oxindole‐3‐acetyl‐aspartate; oxIAA‐GE, 2‐oxindole‐3‐acetyl‐1‐glucosyl ester.

Since the understanding of the ability of plant cell lines to proliferate without supplied auxin (auxin‐habituation) is still elusive, we performed a detailed characterization of two pairs of exponentially growing lines that were independently derived from two different tobacco varieties, and also independently habituated, namely BY‐2/BY‐2H and VBI‐0/VBI‐2 (Fig. 1a). To reveal whether the proliferation of the habituated lines is still dependent on internally synthesized IAA, we first analyzed auxin metabolism and signaling. Levels of endogenous IAA and its metabolites were detected by LC‐MS in all tested cultures (Fig. 1b). The total levels of all IAA metabolites were several times higher in both VBI lines than in both BY‐2 lines, with the highest abundance detected in the auxin‐dependent VBI‐0 cell line (*c*. 500 pmol g^−1^ FW), while *c*. 50 times lower levels were detected in the auxin‐dependent BY‐2 cell line (10 pmol g^−1^ FW). The most abundant metabolite in VBI‐0 and VBI‐2 cells was oxIAA‐GE (360 and 170 pmol g^−1^ FW, respectively).

Habituated VBI‐2 cells growing without exogenous auxin showed *c*. twofold lower levels of total IAA metabolites and *c*. threefold lower levels of free IAA compared with auxin‐dependent VBI‐0. In contrast to VBI lines, the habituated BY‐2H line growing without exogenous auxin showed *c*. 4.5‐fold higher levels of total IAA metabolites than auxin‐dependent BY‐2, with significantly higher (student *t*‐test *P*‐value ≤ 0.05, fold change ≥ 2) levels of IAA, IAA‐GE, oxIAA‐GE and oxIAA‐Asp with the last two being the most abundant auxin metabolites in BY‐2H cells (Fig. 1b). Interestingly, contrary to the dramatic difference in IAA and its metabolites in the auxin‐dependent BY‐2 and VBI‐0 lines, levels of free IAA were very similar in both habituated lines BY‐2H and VBI‐2.

To test whether the cell proliferation independent of exogenous auxin is related to IAA biosynthesis, we treated the cell lines simultaneously with two inhibitors of auxin biosynthesis Yucasin DF and L‐kynurenine (He *et al*., 2011; Tsugafune *et al*., 2017). Surprisingly, the treatment with 20 μM Yucasin DF and 2 μM L‐kynurenine had no significant effect on the proliferation of cell suspension and weight gain of calli (Figs 1c, S1, S2). In addition, the endogenous content of IAA and its metabolites was changed only very little upon treatment with Yucasin DF/l‐kynurenine in all treated samples (Fig. 1d). The only significant decrease was observed for oxIAA in all cell lines and oxIAA‐GE in VBI‐0 cells.

Altogether, our data indicate active IAA biosynthesis and metabolism of variable intensity among the tested lines. Relatively low, but consistently detectable levels of free IAA in the habituated BY‐2H and VBI‐2 cells suggest their auxin‐autonomous proliferation.

### IAA feeding experiments indicate higher uptake of IAA in auxin‐habituated lines and their variable resistance to auxin signaling inhibitors

**Fig. 2:**
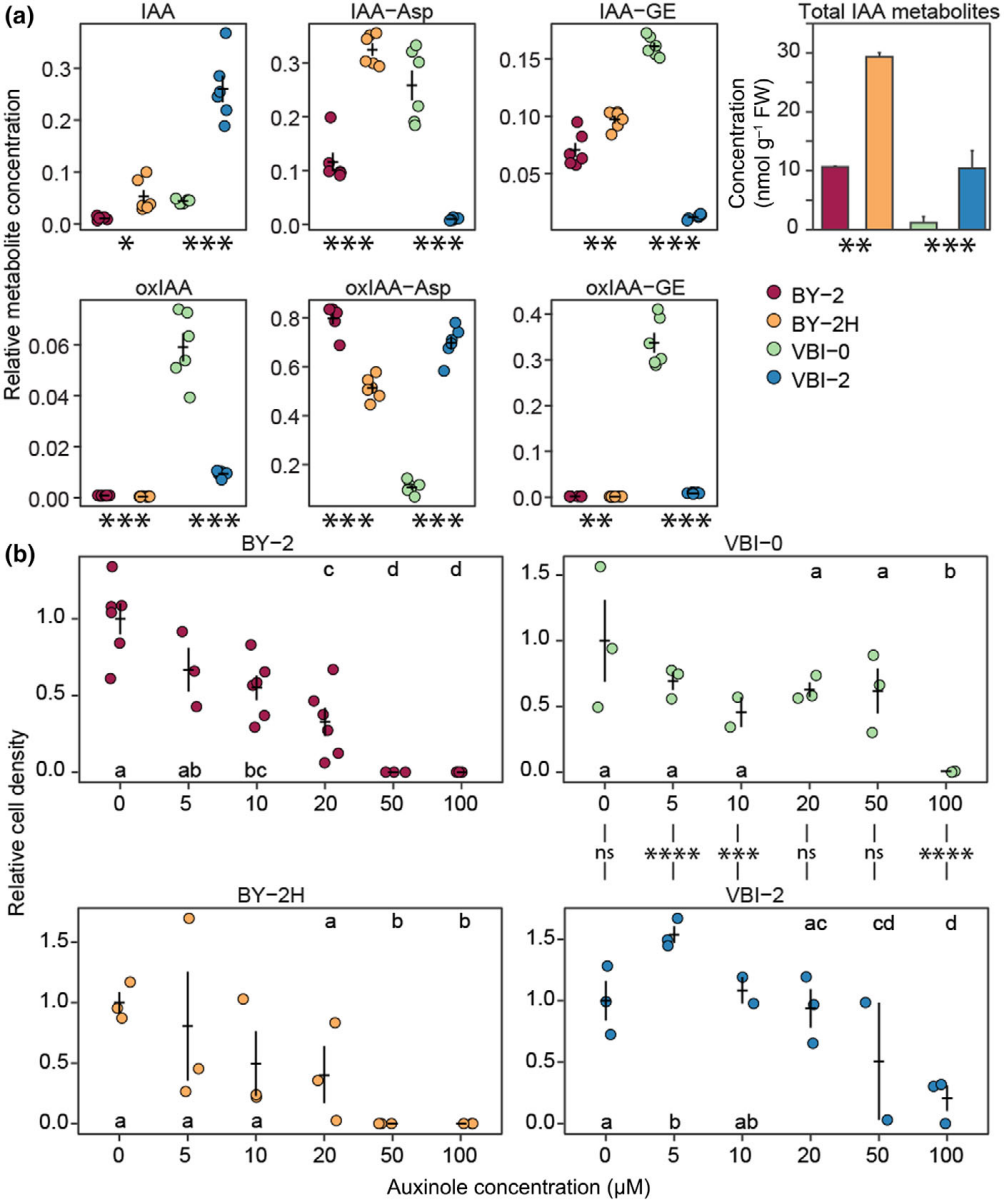
Uptake and metabolism of exogenously applied indole‐3‐acetic acid (IAA) and auxin signaling inhibitor treatments. (a) Relative levels of IAA metabolites in tobacco cells in exponential phase of the growth 2 h after 1 μM IAA treatment. Values (data points and means ±SE) are related to the total content of IAA metabolites in a particular cell line. The significance of the difference in BY‐2H vs BY‐2 and VBI‐2 vs VBI‐0 comparisons was tested by Student's *t*‐test. Error bars indicate average metabolite concentration ±SE. The bar chart represents values of the sum of concentration of IAA and all detected IAA metabolites. (b) Effect of auxinole treatment on the growth of tobacco cell cultures. Data points represent mean cell density from 10 aliquots related to the controls. Error bars indicate average cell density ±SE. Statistical differences among auxinole concentration groups (lowercase letters) were assessed using the Kruskal–Wallis test followed by Dunn's *post hoc* test, with the significance level adjusted using the Bonferroni correction. Statistical significance between cell lines (BY‐2 vs BY‐2H and VBI‐0 vs VBI‐2 pairs) at each auxinole concentration was evaluated using an unpaired Welch's *t*‐test with Bonferroni correction. For the BY‐2 pair, no significant difference (ns) was found. The number of asterisks indicates *P*‐value, *P*‐values: *, *P* < 0.05; **, *P* < 0.01; ***, *P* < 0.001; ****, *P* < 0.0001. IAA‐Asp, indole‐3‐acetyl‐aspartate; IAA‐GE, indole‐3‐acetyl‐1‐glucosyl ester; oxIAA, 2‐oxindole‐3‐acetic acid; oxIAA‐Asp, 2‐oxindole‐3‐acetyl‐aspartate; oxIAA‐GE, 2‐oxindole‐3‐acetyl‐1‐glucosyl ester.

To compare the uptake and metabolism of IAA in auxin‐habituated lines, we performed a quantitative analysis of IAA and its metabolites by LC‐MS in cells treated with 1 μM IAA for 2 h. The total amount of internalized IAA and its metabolites was significantly higher in both auxin‐habituated lines than in their auxin‐dependent counterparts (Fig. 2a).

Interestingly, in both auxin‐habituated lines, free IAA represented several times a higher proportion of the total IAA content than their auxin‐dependent counterparts, suggesting their decreased IAA metabolism. We also noted that BY‐2 and BY‐2H lines showed overall higher uptake and metabolism of added IAA when compared to VBI‐0 and VBI‐2. The most abundant metabolite of IAA was oxIAA‐Asp in BY‐2, BY‐2H and VBI‐2 cells. VBI‐0 cells metabolized IAA mostly into IAA‐Asp, IAA‐GE and oxIAA‐GE.

To test the importance of the canonical auxin signaling pathway for cell proliferation in auxin‐habituated lines, we further quantified cell densities after treatments with increasing concentrations of competitive inhibitors of auxin signaling, auxinole and PEO‐IAA (Hayashi *et al*., 2012). As shown in Fig. 2(b), the effect of auxinole on the proliferation of the habituated BY‐2H line was comparable to BY‐2. In the case of VBI lines, the habituated VBI‐2 showed higher resistance in the most auxinole concentrations. We further confirmed this higher resistance by PEO‐IAA treatments (Fig. S3).

Altogether, these results suggest that BY‐2H and VBI‐2 retained and even enhanced the ability to uptake and metabolize IAA. The importance of auxin signaling for both habituated and nonhabituated lines was also indicated by the inhibitory effects of auxinole and PEO‐IAA on their proliferation.

### Transcriptome analysis indicates a different mechanism of auxin‐habituation in BY‐2H and VBI‐2

**Fig. 3:**
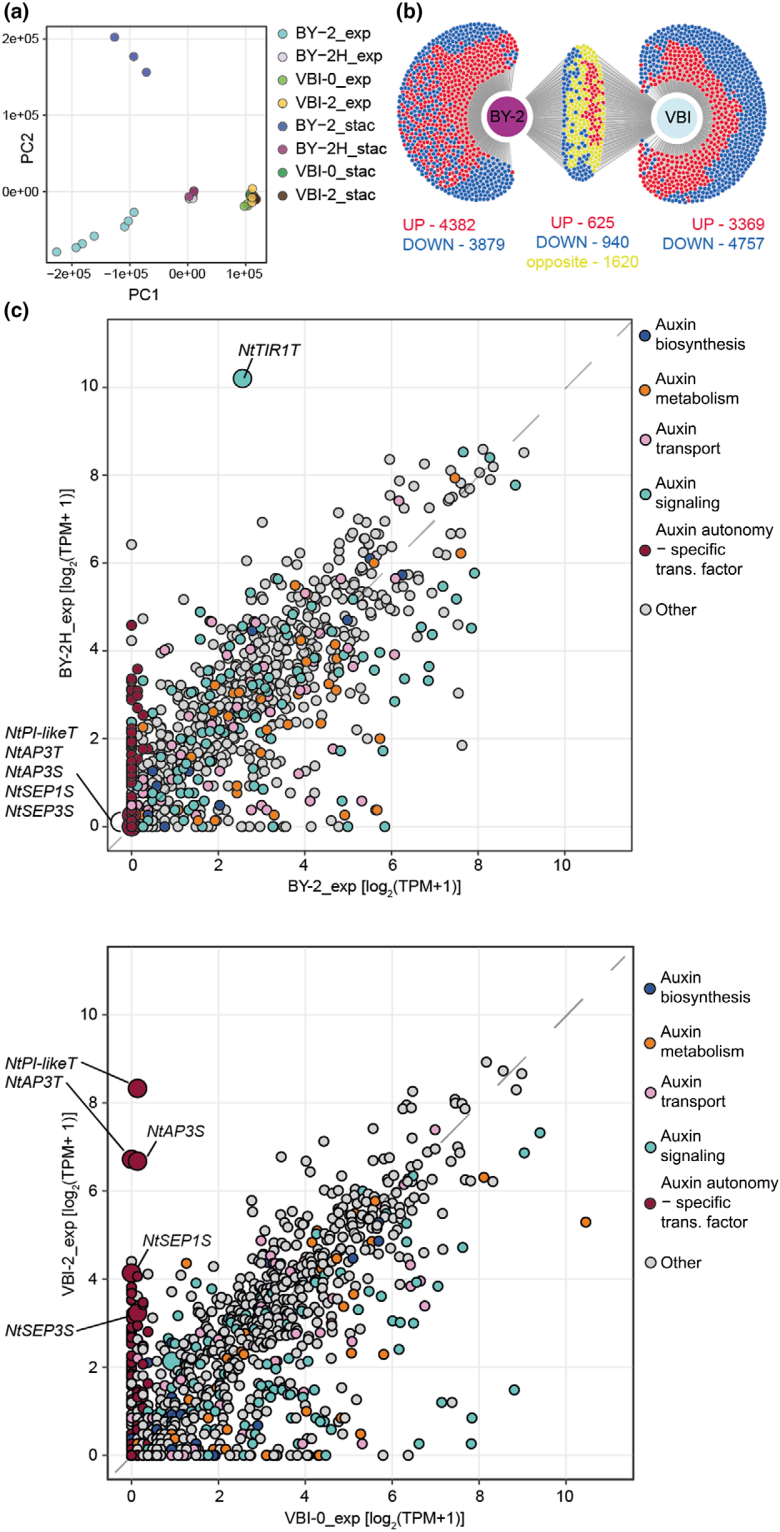
Transcriptome comparisons between exponential (exp) and stationary (stac) auxin‐habituated (BY‐2H, VBI‐2) and auxin‐dependent (BY‐2, VBI‐0) tobacco cell lines. (a) Projections of RNA‐seq data onto principal components (PC). Axes labels show values of principal components calculated from sleuth‐normalized est_count units. (b) DiVenn diagram visualizing comparison between lists of transcripts differentially regulated in BY‐2H vs BY‐2 and VBI‐2 vs VBI‐0. (c) Scatter plots representing a comparison between transcript levels (TPM – transcripts per million) of auxin‐related genes in exponential stages BY‐2H vs BY‐2 and VBI‐2 vs VBI‐0. Large circles represent genes with putative roles as drivers of auxin autonomy in BY‐2H (NtTIR1T) and VBI‐2 (MADS‐box TFs).

To reveal the mechanism behind cell proliferation independent of externally supplied auxin, we performed transcriptome analysis of all tested tobacco cell lines harvested at their exponential and stationary phases. RNA quality and total number of reads per biological replicate are summarized in Table S2. After quality filtering, reads were mapped against the *Nicotiana tabacum* v.4.5 cDNA reference dataset (Edwards *et al*., 2017) using the Salmon software (Patro *et al*., 2017). Principal component analysis and Jensen–Shannon divergence scores indicated a clear distinction of samples within their respective groups and established a solid basis for conducting further data analysis and for performing comparisons between samples (Figs 3a, S4). Transcripts with an average TPM value greater than 0.5 in at least one sample variant were considered detected (50 241 of 69 491 transcripts in the reference dataset). A table showing full results of RNA‐seq analysis is provided as Dataset S1.

To examine whether BY‐2H and VBI‐2 cell lines share a similar mechanism of auxin autonomy, we looked for the overlap between groups of genes differentially expressed in BY‐2H cells compared with BY‐2, and VBI‐2 cells compared with VBI‐0. First, we identified 11 446 genes with the same differential regulation in both phases of the BY‐2H sub‐cultivation cycle compared with BY‐2 cells (5824 upregulated and 5622 downregulated). Similarly, 11 311 DEGs were identified between VBI‐2 and VBI‐0 cells (4797 upregulated and 6514 downregulated in both phases). The overlap of these groups of DEGs consisted of 625 and 940 genes, which were upregulated and downregulated, respectively, in both BY‐2H and VBI‐2 cell lines compared with their auxin‐dependent variants. Interestingly, 1620 genes showed the opposite regulation in this comparison (Fig. 3b). These gene sets were analyzed using the PANTHER overrepresentation assay, which revealed that no GO term was overrepresented within the upregulated genes and that processes related to ATP biosynthesis, homeostasis and glutathione metabolism were overrepresented in the downregulated genes in both VBI‐2 and BY‐2H cell lines (Table S3).

Altogether, the overlap between sets of genes differentially expressed in the two auxin‐habituated lines was not convincing and there was also no obvious enrichment in genes related to auxin signaling, metabolism or transport. Therefore, our data strongly suggest that the mechanisms of auxin autonomy differ in the two auxin‐habituated cell lines.

### Auxin receptor TIR1 and MADS‐domain transcription factors are upregulated in auxin‐autonomous cells

To get a deeper insight into the mechanism of habituation in the two auxin‐autonomous cell lines, we examined the transcriptomic data specifically for TFs and genes related to auxin homeostasis and auxin signal transduction. Genes from the auxin‐related list were classified into categories related to auxin metabolism (biosynthesis, inactivation and reactivation), auxin transport (efflux, influx and endomembrane transport) and auxin perception (*TIR1/AFB*s, *AUX/IAA*s and *ARF*s; Table S4). Levels of these transcripts were correlated in the pairs of exponentially growing auxin‐autonomous and auxin‐dependent lines BY‐2H vs BY‐2 and VBI‐2 vs VBI‐0 (Fig. 3c). In BY‐2H cells, the gene coding for the homolog of the auxin receptor TIR1 (*NtTIR1T*) was the 10th most upregulated gene in the dataset, exhibiting *c*. 30‐fold higher transcript abundance in BY‐2H than in BY‐2. Among other upregulated genes related to auxin, we found homologs of genes involved in auxin biosynthesis *AMI1* and *TAA1*. In VBI‐2 cells, we observed no remarkable effect on auxin homeostasis as upregulated genes were usually accompanied by similar levels of downregulated genes with the same function with the exception of genes related to auxin conjugation (e.g. *NtUGT3* and GH3s), which were present at remarkably higher levels in both auxin‐dependent cell lines than in their auxin‐habituated counterparts (1 upregulated vs 16 downregulated genes in BY‐2 and 0 upregulated vs 16 downregulated genes in VBI; Table S5).

Regarding TFs, a simple criterion of upregulated (*q*‐value ≤  0.05, fold change ≥  2) in auxin‐autonomous cells yielded *c*. 400 transcripts; therefore, we applied more stringent criteria to select only strong candidates, which might be more likely responsible for driving proliferation of the auxin‐habituated lines. The resulting list contained 72 genes (Table S6), of which 27 and 50 were specific to BY‐2H and VBI‐2 cells, respectively. Only five genes were upregulated in both habituated lines, highlighting different strategies of auxin habituation. Transcript levels of the 10 most abundant in BY‐2H and VBI‐2 cells are shown in Figs S5 and S6, respectively. The most upregulated TFs in VBI‐2 cells were homologs of MADS family proteins APETALA3, PISTILLATA and SEPALLATA1 (Fig. 3c), which are naturally expressed in floral organs and are repressed by the POLYCOMB REPRESSIVE COMPLEX 2 (PRC2) in vegetative tissues (Kim *et al*., 2010). Interestingly, we found that EMBRYONIC FLOWER 2 (EMF2), a component of this ‘vegetative type’ of PRC2 is strongly downregulated in VBI‐2 cells (Table S7). By contrast, in BY‐2H cells, the most transcribed line‐specific TFs were homologs of AGAMOUS‐like, MYB4‐like and ethylene‐responsive TFs, although their transcription levels were remarkably lower than the most abundant TFs in VBI‐2 cells.

These results strongly point to the completely different mechanisms of auxin habituation between the two habituated lines.

### Auxin receptor NtTIR1T is strongly upregulated in BY‐2H as a result of extensive gene multiplication

**Fig. 4:**
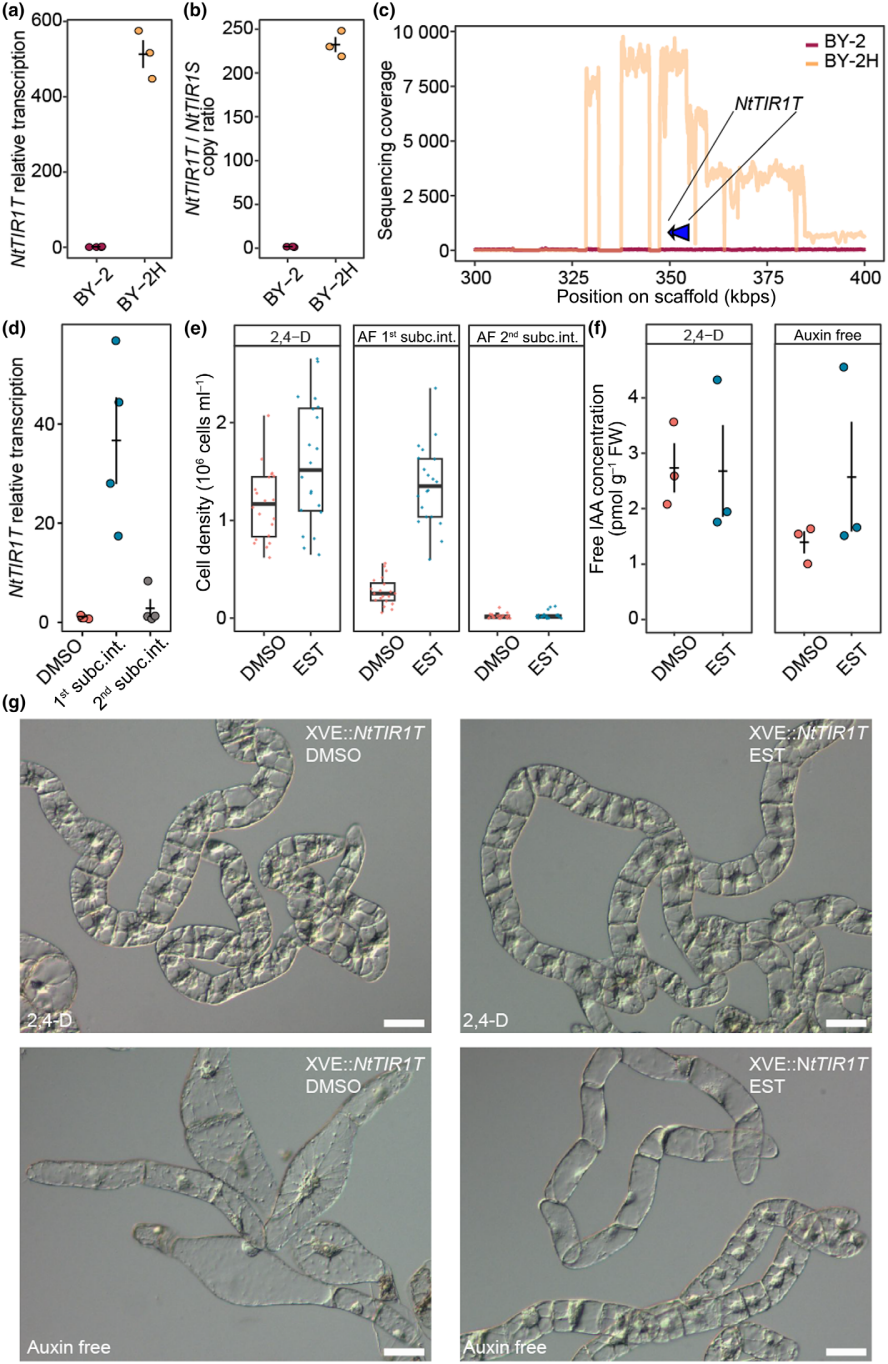
Evidence for *NtTIR1* gene multiplication in BY‐2H and auxin‐autonomous growth upon its overexpression in BY‐2 tobacco cells. (a) RT‐qPCR of *NtTIR1* in BY‐2 and BY‐2H cell lines. Error bars indicate average relative transcript levels ±SE. (b) Gene copy ratio of *NtTIR1T* and *NtTIR1S* determined by qPCR. Error bars indicate average gene copy ratio ±SE. (c) Sequence coverage of *NtTIR1T* gene obtained from whole‐genome sequencing data of BY‐2 and BY‐2H cells mapped against tobacco reference. Blue arrow indicates *NtTIR1T* region. (d) The relative transcription of *XVE::NtTIR1T* in noninduced (dimethyl sulfoxide (DMSO)) and induced (β‐estradiol, EST) BY‐2 cells in two subsequent subculture intervals (subc.int.). Error bars indicate average relative transcript levels ±SE. (e) Cell density in 2‐d‐old induced and noninduced *XVE::NtTIR1T* cells in control and auxin‐free (AF) media, determined in two subsequent subculture intervals. Boxplots show median, first and third quartiles, and the whiskers extend to the largest and smallest measured values. (f) Levels of endogenous free indole‐3‐acetic acid (IAA) in 2‐d‐old transgenic *XVE::NtTIR1T* cells in the first subculture interval. Error bars indicate average free IAA concentration ±SE. (g) Phenotype of *XVE::NtTIR1T* cells in control and auxin‐free media, differential interference contrast microscopy. Induction of *NtTIR1T* phenocopies the effect of 2,4‐dichlorophenoxy acetic acid (2,4‐D), whereas DMSO‐treated cells show a typical auxin‐starvation phenotype. Bars, 50 μm. TIR1, TRANSPORT INHIBITOR RESPONSE 1.

Since the overexpression of *NtTIR1T* was a very promising candidate to induce auxin habituation of BY‐2H cells, we decided to analyze the background of its very high transcription levels. First, we verified the upregulated transcription of *NtTIR1T* by RT‐qPCR (Fig. 4a). We also confirmed *Nt*TIR1T upregulation at the protein level by mass spectrometry. *Nt*TIR1T in BY‐2H cells was the 398th most abundant protein out of more than 6000 identified proteins, exceeding the detection threshold more than 50‐fold. In the BY‐2 proteome, *Nt*TIR1T was not detected at all (Table S8). Next, we compared the promoter sequences of *NtTIR1* in BY‐2 and BY‐2H. They were identical, but the two reactions strongly differed in the PCR product level. Therefore, we performed quantitative PCR on DNA isolated from BY‐2 and BY‐2H cells using gene‐specific primers for *NtTIR1T* and its homolog *NtTIR1S*. The ratio of gene copy numbers was calculated using the equation for relative quantification. While the *NtTIR1T*/*NtTIR1S* ratio was *c*. 1 in BY‐2 cells, in BY‐2H, we found this ratio to reach a value of 230, which indicated massive multiplication of the gene in BY‐2H cells (Fig. 4b).

To verify the *NtTIR1T* gene multiplication, we performed whole‐genome DNA resequencing of BY‐2 and BY‐2H lines. We obtained 1.58 billion reads (almost 240 Gbps) for BY‐2 and 1.3 billion reads (almost 200 Gbps) for BY‐2H. Mapping of reads to the *Nicotiana tabacum* reference genome (Nitab‐v4.5_genome_Scf_Edwards2017) was done by bwa, and coverage of the *NtTIR1T* gene neighborhood was determined by samtools. Coverage of the region coding for *NtTIR1T* (scaffold Nitab4.5_0000824, pos 349 583–354 771 bps) was < 100× in BY‐2 and between 8000× and 9000× in the BY‐2H sample (Fig. 4c). Surprisingly, varying coverage near *NtTIR1T* in BY‐2H indicates that the multiplication occurred in several rounds with genomic regions of at least three different lengths, of which the longest is *c*. 142 kbp long. We also analyzed whether the multiplied *NtTIR1T* sequence was a subject of mutations, which might be positively selected in the cell culture to achieve altered auxin sensing. However, a detailed analysis of sequencing data revealed no change in the coding sequence (SNPs or indels) in any copy of the gene, indicating that the auxin autonomy was connected with the *NtTIR1T* upregulation itself.

Altogether, our data show that the increase in *NtTIR1T* expression was not driven by altered sequence or epigenetic labeling of the *TIR1* promoter as we expected, but by unusual multiplication of the *TIR1* chromosomal region.

### Overexpression of *NtTIR1T* and auxin biosynthetic genes in BY‐2 cells results in auxin‐autonomous proliferation

To test the role of TIR1 in auxin‐autonomous cell proliferation, we generated β‐estradiol‐inducible BY‐2 line *XVE::NtTIR1T*. We selected two independent lines with the highest *NtTIR1T* overexpression, showing approximately a 40‐fold increase in *NtTIR1T* transcription after induction compared with noninduced controls (Fig. 4d). The performance of the cells was monitored in the 2‐d‐old culture by quantification of cell density and by comparing the cell phenotypes microscopically. No difference was observed in the 2,4‐D‐supplemented samples between β‐estradiol‐treated and DMSO‐treated cells. However, in the absence of exogenous auxin, the noninduced cells showed all symptoms of auxin starvation and did not proliferate in contrast to induced cells, which showed no signs of auxin deficiency and whose proliferation activity was the same as that of the auxin‐supplemented cells (Fig. 4e,g). However, when we continued the cultivation of induced cells for one more subculture interval, the transcript levels of *NtTIR1T* were not increased likely due to rapid silencing of its expression (Fig. 4d). Although undesirable, this observation clearly validates the correlation between high *NtTIR1T* expression and cell proliferation independent of external auxin.

To determine whether the high expression of *NtTIR1T* is associated with higher levels of free IAA, we analyzed the levels of auxin metabolites and found no significant differences in all sample groups examined (Fig. 4f). Altogether, we conclude that high expression of the auxin receptor *Nt*TIR1T is sufficient for auxin‐autonomous proliferation of BY‐2 cells. To test whether the induction of auxin biosynthesis would also cause independence of the external auxin, we also prepared β‐estradiol‐inducible BY‐2 lines carrying two biosynthetic enzymes XVE::*NtTAA1* + *NtYUCCA3*. As expected, β‐estradiol‐treated cells of these lines showed no signs of auxin starvation and standard proliferation thanks to significantly increased IAA levels in comparison with DMSO‐treated culture (Fig. S7).

In summary, both increased IAA biosynthesis and enhanced sensitivity to IAA due to *TIR1* overexpression in the nonhabituated BY‐2 cells induced their proliferation even in the auxin‐free media.

## Discussion

### Regulation of cell proliferation by auxin *in planta* and in cell lines – different ways leading to the same result

Auxin is often described as a plant morphogen influencing plant body shape and development. The precise regulation of auxin action depends on its biosynthesis, metabolism and transport as well as on the cell‐specific context of the signal perception and transduction machinery. The evidence in plants shows that local synthesis of IAA is important for maintaining auxin maxima and the meristematic potential of cells in the quiescence centre of roots (Brumos *et al*., 2018; Cao *et al*., 2019). On the contrary, IAA biosynthesis does not change in response to wounding connected with callus induction, in contrast to elevated cytokinin levels (Ikeuchi *et al*., 2017). Elevated levels of auxin and cytokinins have been published in *Agrobacterium*‐induced tumors as well as in tumors of inbred *Nicotiana* lines or interspecies hybrids (reviewed in Dodueva *et al*., 2020). It shows that precise regulation of both auxin and cytokinin maxima enables strict control of cell proliferation sites *in planta*, preventing the development of tumors.

Unlike in tumors, proliferation of normal cells within the plant body is mostly dependent on phytohormonal and other stimuli from the surrounding cells. This is also reflected by the dependence of the *in vitro* cultured cell lines' proliferation on exogenously supplied phytohormones. An exact combination of auxins and cytokinins has to be used to establish cell lines. However, cytokinin dependence can be later easily abolished by a reproducible and reversible habituation process. It was shown that this process is based on the cytokinin deficiency‐induced modulation of gene expression accompanied by epigenetic reprogramming of the habituated culture. This can eventually lead to an increased expression of the cytokinin receptor AHK4, which is considered to be the main factor in cytokinin habituation (Pischke *et al*., 2006). In contrast to cytokinins, inducing auxin habituation simply by auxin starvation is rarely successful and appears hardly reproducible or reversible. Several studies have achieved auxin habituation by introducing a Ti plasmid from Agrobacterium, which carries *iaaM* and *iaaH* genes for IAA synthesis, or by physical or chemical mutagenesis (Binns & Meins, 1973; Noguchi *et al*., 1977; Binns *et al*., 1982, 1987; Campell & Town, 1991; Campell *et al*., 1992; Frank *et al*., 2000). Analysis of tobacco cell line 2B‐13 derived from BY‐2 line by long auxin starvation and UV irradiation (Noguchi *et al*., 1977) suggested that auxin habituation was connected with the presence of an extracellularly secreted factor CDF (Shimizu *et al*., 2006). Unfortunately, the habituated line was lost before providing functional evidence and the mechanism behind it (Nagata, 2023).

In the BY‐2H cell line analyzed in this study, the auxin autonomy seems to be primarily connected with increased auxin sensitivity resulting from enhanced expression of the *NtTIR1T* gene. The analysis of the BY‐2H genome revealed that the upregulation was caused by a massive multiplication of the *NtTIR1T* genomic region. The mechanism of amplification remains unclear and is a subject of the follow‐up research. Anyhow, as with most previously published studies on auxin habituation, the independence of exogenous auxin was a direct consequence of the altered genomic sequence of the habituated BY‐2H line.

In VBI‐2 cell line, we suppose that the auxin autonomy might be connected with altered epigenetic regulation by the PRC2. We hypothesize that upregulation of several MADS‐box gene homologs (*APETALA3*, *SEPALLATA3* and *PISTILLATA*) was caused by decreased expression of a PRC2 component EMF2, which is known to be involved in repression of these genes in *Arabidopsis thaliana* (Kim *et al*., 2010, 2012). Although the primary cause of the altered EMF2 expression might also result from mutation, the possible connection of auxin autonomy with upregulation of the set of flowering‐specific MADS‐box genes is an exciting hypothesis that is currently under investigation.

In summary, our results on the molecular mechanism of auxin autonomy on two independently habituated tobacco cell lines clearly show that, unlike cytokinin habituation, auxin autonomy is not a universally reproducible phenomenon and that there is no common mechanism of auxin habituation. As a proof of concept, we also succeeded in inducing auxin autonomy in BY‐2 cells by overexpression of auxin biosynthetic genes *NtTAA1T* and *NtYUC3T*. It shows another possible mode of auxin habituation which might be ‘naturally’ achievable by mutations in the regulatory regions of these genes. The habituation by either increased auxin biosynthesis or auxin sensing might present an appealing option for biotechnological use of cell lines.

### Overcoming the auxin starvation by enhancing auxin perception

Plant cell proliferation has long been considered dependent on auxin. However, as recently shown by Prigge *et al*. (2020), Arabidopsis zygote with knocked out all TIR1/AFB paralogs can undergo cell divisions even without the functional canonical auxin signaling pathway (Prigge *et al*., 2020; Shimotohno *et al*., 2021). In both lines analyzed here (BY‐2H and VBI‐2), cell proliferation was still dependent on auxin, although the mechanism of auxin autonomy differed. The auxin habituation of the BY‐2H line seems to result from the highly enhanced expression of the auxin TIR1 receptor gene. Our attempts to confirm it directly by silencing *NtTIR1T* in the BY‐2H line failed, but overexpression of the gene in BY‐2 induced proliferation independent of external auxin, which is strongly supportive of our hypothesis. The effect of *NtTIR1T* overexpression in both lines shows a highly interesting phenomenon that an increased number of auxin receptors are able to compensate to a certain extent for lower auxin availability. Although the intracellular concentration of free IAA is roughly comparable in BY‐2H and BY‐2 cells, the external 2,4‐D is completely missing in the case of BY‐2H. A similar mechanism of increased auxin sensitivity promoting a stronger response has been published in the context of stress reaction. Plants overexpressing auxin receptors were shown to have higher salt tolerance due to upregulated downstream auxin‐responsive genes (Xie *et al*., 2000; Yang *et al*., 2013; Chen *et al*., 2017; Salehin *et al*., 2019; Marzi *et al*., 2024). The observed effect of enhanced signaling due to higher receptor concentration can be explained by the shift in the thermodynamic equilibrium between free IAA and free TIR1 toward IAA bound to its receptor. The signal strength can be influenced by the concentration of each ‘reactant’, so a higher level of the receptor can mimic the effect of a higher level of the ligand (Goldbeter & Wolpert, 1990; Wend *et al*., 2013).

The interpretation of the effects of increased TIR1 levels is even more complicated when we consider the recently published activity of auxin receptors as producers of the cyclic adenosine monophosphate serving as second messengers in addition to the degron‐based signaling system (Qi *et al*., 2022, 2023). Also, recently, the link between the reactive oxygen species signaling pathway and auxin receptors was published. It was shown that FERONIA‐mediated oxidation influences the subcellular localization of TIR1 and AFB2 and thus their activity (Lu *et al*., 2024). With new discoveries, the auxin signaling pathway is becoming increasingly complex, requiring new approaches and further experiments.

### Microevolution in a flask with potential biotechnological applications

The auxin habituation in BY‐2H is also very interesting regarding the process itself of gaining independence from externally supplemented auxin. The amplification of the TIR1 region seems to be a consequence of in‐flask microevolution based on random genomic changes in conjunction with high selection pressure. It is possible that the amplification is still a running process since cells carrying higher TIR1 levels are likely positively selected thanks to a higher proliferation rate. The amplification of the 100‐kbp region containing the *NtTIR1T* gene is not comparable to any previously published data in plants. The phenomenon of microevolution in the flask has been observed in yeast, where changes in cell morphology and culture behavior have been documented after 600 generations of anaerobic stress (Bozdag *et al*., 2023) and even the ability to generate *de novo* genes from noncoding sequences has been shown in this species (Blevins *et al*., 2021; Parikh *et al*., 2022). However, the amplification of the genomic region with *NtTIR1T* in BY‐2H most closely resembles the bacterial response to rare environments, where gene amplification is an alternative way to increase the expression level of specific genes (Tomanek *et al*., 2020). A parallel may also be found in insect genomes, where duplications (amplifications) of genes for detoxification enzymes contribute to resistance to insecticides (Muthu Lakshmi Bavithra *et al*., 2023).

Interestingly, the *NtTIR1T* gene amplification was not counteracted by transcriptional or posttranscriptional silencing. Understanding the reasons behind this process and replicating it could find application in plant biotechnology. Also, the auxin habituation achieved by increased sensing or biosynthesis is of great interest for biotechnology applications, where the elimination of the harmful synthetic auxin could represent an interesting improvement in the plant cell culture cultivation.

## Competing interests

None declared.

## Author contributions

PJ and KM wrote the manuscript, planned experiments and processed data, as well as performed the majority of experiments. EK, ZV, LH and AB participated in cloning, maintained the cell cultures, collected samples and transformed the plant cells. PID and RV performed analytical determinations of auxin metabolites. AP participated in data analysis. MČ made phenotypical evaluations of the transformed cell lines. LF and JP reviewed the manuscript and participated in experimental design and planning. PJ and KM contributed equally to this work.

## Disclaimer

The New Phytologist Foundation remains neutral with regard to jurisdictional claims in maps and in any institutional affiliations.

## Supporting information


**Dataset S1** Complete dataset of transcript abundances (TPM) and results of statistical evaluation (Sleuth algorithms).

**Fig. S1** Effect of combined treatments with Yucasin DF and l‐kynurenine on the growth of calli.
**Fig. S2** Effects of combined treatments with Yucasin DF and l‐kynurenine on the growth of cell suspensions.
**Fig. S3** Effect of PEO‐IAA treatment on cell growth.
**Fig. S4** Heatmap displaying Jensen‐Shannon divergence coefficients for individual RNA‐seq sample comparisons.
**Fig. S5** Transcript levels (ln TPM) of top 10 most abundant transcription factors specific to BY‐2H.
**Fig. S6** Transcript levels (ln TPM) of top 10 most abundant transcription factors specific to VBI‐2.
**Fig. S7** Inducible overexpression of *NtTAA1* and *NtYUCCA3* allow auxin‐autonomous cell proliferation in BY‐2 cells.

**Table S1** List of primers for qPCR and NCBI identifiers of their target.
**Table S2** List of RNA samples used in transcriptome analysis.
**Table S3** List of significantly overrepresented GO terms (Biological Process) in selected differentially expressed transcripts.
**Table S4** List of auxin‐related genes.
**Table S5** Summary of gene classification in autonomous vs dependent line comparisons.
**Table S6** List of transcription factors and transcription regulators specific to auxin‐autonomous cell lines.
**Table S7** List of putative components of POLYCOMB REPRESSIVE COMPLEX (PRC2).
**Table S8** Identification and quantification of NtTIR1 protein by LC‐MS–MS analysis in extracts from 2‐d‐old BY‐2 and BY‐2H cells.
Please note: Wiley is not responsible for the content or functionality of any Supporting Information supplied by the authors. Any queries (other than missing material) should be directed to the *New Phytologist* Central Office.

## Data Availability

Raw RNA‐seq data are available through GEO Series accession nos.: GSE160438 and GSE275968 at the Gene Expression Omnibus (https://www.ncbi.nlm.nih.gov/geo/). The data that support the findings of this study are included as part of the article or in Dataset S1, Figs S1–S7, and Tables S1–S8.
