# Supplementary material for: A novel mechanism of auxin habituation: upregulation of auxin receptor TRANSPORT INHIBITOR RESPONSE 1 allows cell proliferation independent of external auxin
Source: New Phytol. 2025 Dec 9;249(3):1268–82. doi: 10.1111/nph.70763 (PMC12780323; doi:10.1111/nph.70763)
Supplement: Supplementary file 2 — Fig. S1 Effect of combined treatments with Yucasin DF and l‐kynurenine on the growth of calli. Fig. S2 Effects of combined treatments with Yucasin DF and l‐kynurenine on the growth of cell suspensions. Fig. S3 Effect of PEO‐IAA treatment on cell growth. Fig. S4 Heatmap displaying Jensen‐Shannon divergence coefficients for individual RNA‐seq sample comparisons. Fig. S5 Transcript levels (ln TPM) of top 10 most abundant transcription factors specific to BY‐2H. Fig. S6 Transcript levels (ln TPM) of top 10 most abundant transcription factors specific to VBI‐2. Fig. S7 Inducible overexpression of NtTAA1 and NtYUCCA3 allow auxin‐autonomous cell proliferation in BY‐2 cells. [file NPH-249-1268-s001.pdf]

New Phytologist

New Phytologist **Supporting Information**

Article title: A novel mechanism of auxin habituation: upregulation of auxin receptor TRANSPORT INHIBITOR RESPONSE 1 allows cell proliferation independent of external auxin
Authors: Pavel Jelínek, Karel Müller, Eliška Kobercová, Adéla Přibylová, Milada Čovanová, Petre I. Dobrev, Roberta Vaculíková, Zuzana Vondráková, Lenka Helusová, Anita Bírošíková, Lukáš Fischer, Jan Petrášek
Article acceptance date: 27 October 2025

The following Supporting Information is available for this article:

Fig. S1 Effects of combined treatments with Yucasin DF and L-kynurenine on the growth of calli.

Fig. S2 Effects of combined treatments with Yucasin DF and L-kynurenine on the growth of cell suspensions.

Fig. S3 Effect of PEO-IAA treatment on cell growth.

Fig. S4 Heatmap displaying Jensen-Shannon divergence coefficients for individual RNA-seq sample comparisons.

Fig. S5 Transcript levels (ln TPM) of top 10 most abundant transcription factors specific to BY-2H.

Fig. S6 Transcript levels (ln TPM) of top 10 most abundant transcription factors specific to VBI-2.

Fig. S7 Inducible overexpression of *NtTAA1* and *NtYUCCA3* allow auxin-autonomous cell proliferation in BY-2 cells.

Table S1 List of primers for qPCR and NCBI identifiers of their target.

Table S2 List of RNA samples used in transcriptome analysis.

Table S3 List of significantly overrepresented GO terms (Biological Process) in selected

differentially expressed transcripts.

Table S4 List of auxin-related genes.

Table S5 Summary of gene classification in autonomous vs dependent line comparisons.

Table S6 List of transcription factors and transcription regulators specific to auxin autonomous cell lines.

Table S7 List of putative components of POLYCOMB REPRESSIVE COMPLEX (PRC2).

Table S8 Identification and quantification of NtTIR1 protein by LC/MS-MS analysis in extracts from 2-day-old BY-2 and BY-2H cells.

Dataset S1 Complete dataset of transcript abundances (TPM) and results of statistical evaluation (Sleuth algorithms).

Methods S1 Click here to enter text.

Notes S1 Click here to enter text.

Video/Movie S1 Click here to enter text.

Fig. S1 Effects of combined treatments with Yucasin DF and L-kynurenine on the growth of calli. Data points represent the fresh weight increase per individual callus after two weeks of treatment. Error bars indicate average weight increase +/- SE. No effect of YucDF+Kyn treatment compared to DMSO treatment was statistically significant (Student t-test).

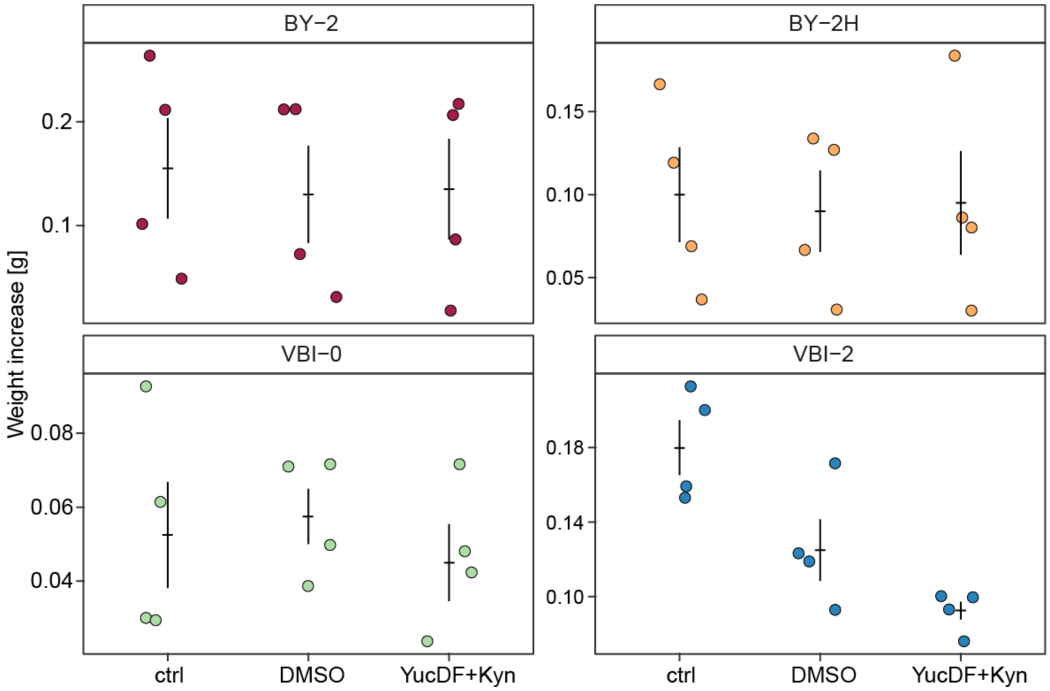

Fig. S2 Effects of combined treatments with Yucasin DF and L-kynurenine on the growth of cell suspensions. Data points represent the mean fresh weight of three 5 mL cell suspension aliquots. No effect of YucDF+Kyn treatment compared to DMSO treatment was statistically significant (Student t-test).

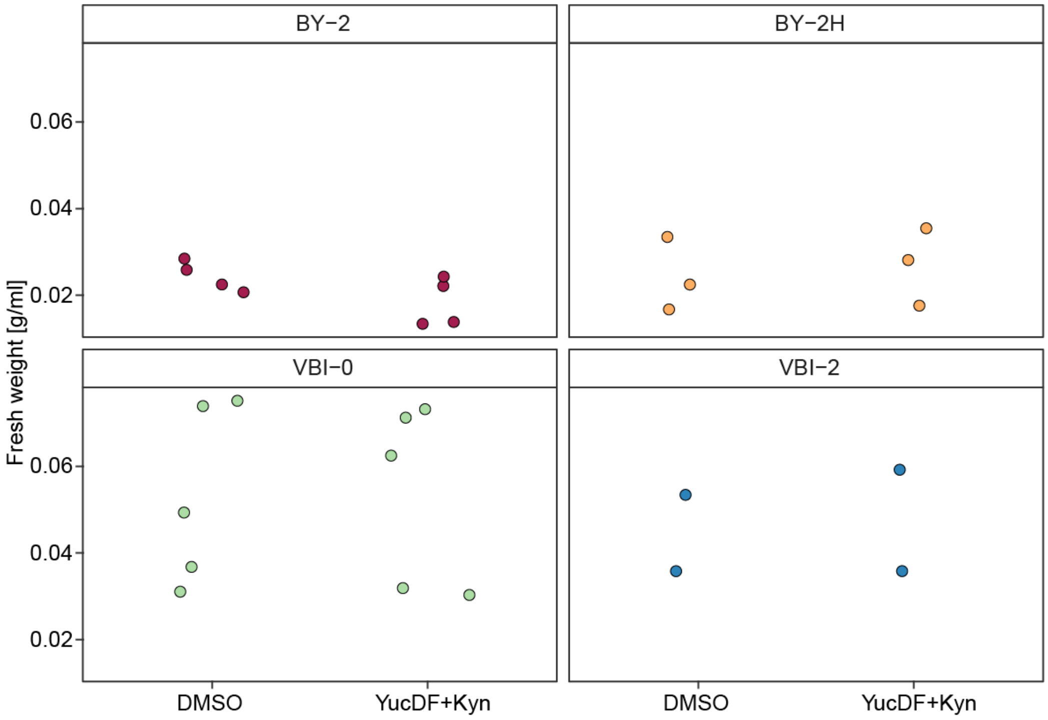

Fig. S3 Effect of PEO-IAA treatment on cell growth. Data points indicate relative cell counts related to the control (DMSO treatment).

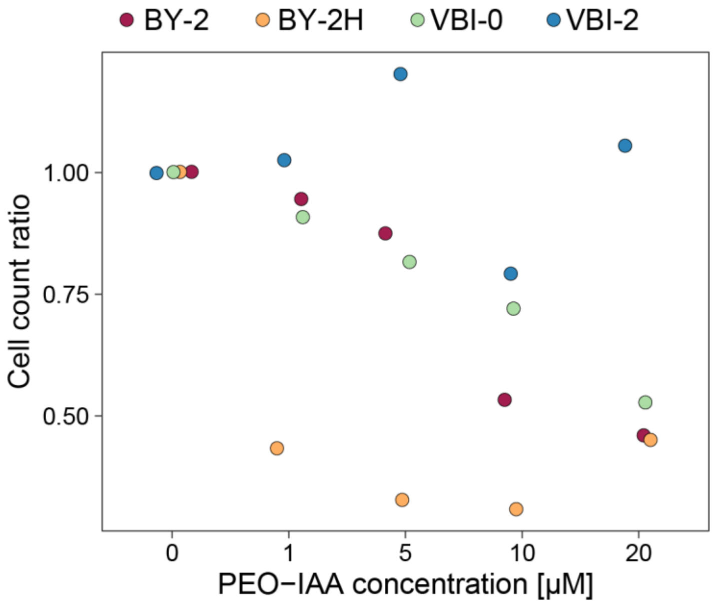

Fig. S4 Heatmap displaying Jensen-Shannon divergence coefficients for individual RNA-seq sample comparisons. RNA was isolated from approximately 50 mg of cell collected in their exponential (2-day-old cells for BY-2, 3-day-old cells for BY-2H, VBI-0 and VBI-2) and stationary (7-day-old for BY-2, 10-day-old for BY-2H, VBI-0 and VBI-2) phases. RNA-seq was performed by service companies GATC-Biotech and Novogene. Levels of transcript abundances were estimated using Salmon. Heatmap was generated by sleuth package in R.

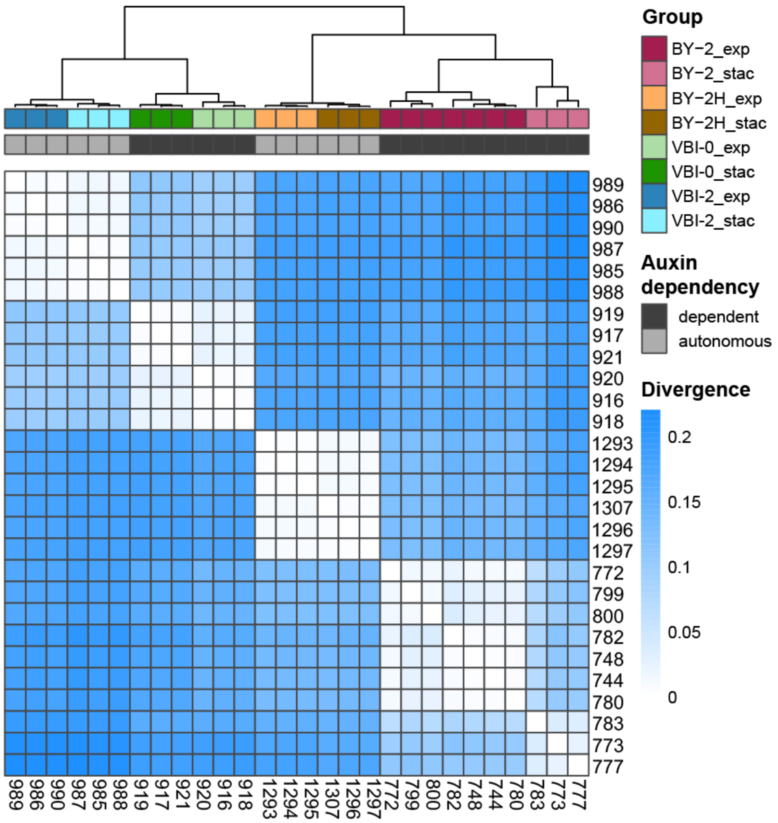

Fig. S5 Transcript levels (ln TPM) of top 10 most abundant transcription factors specific to BY-2H. Transcription factors in tobacco transcriptome dataset was first filtered to select only those specific to auxin habituated cell lines (TPM in all BY-2 and VBI-0 samples <= 0.5 AND TPM in all BY-2H and VBI-2 samples >= 1). Transcription of ten most abundant TFs in BY-2H_exp is shown in heatmap. Table **S6** contains list of all filtered TFs.

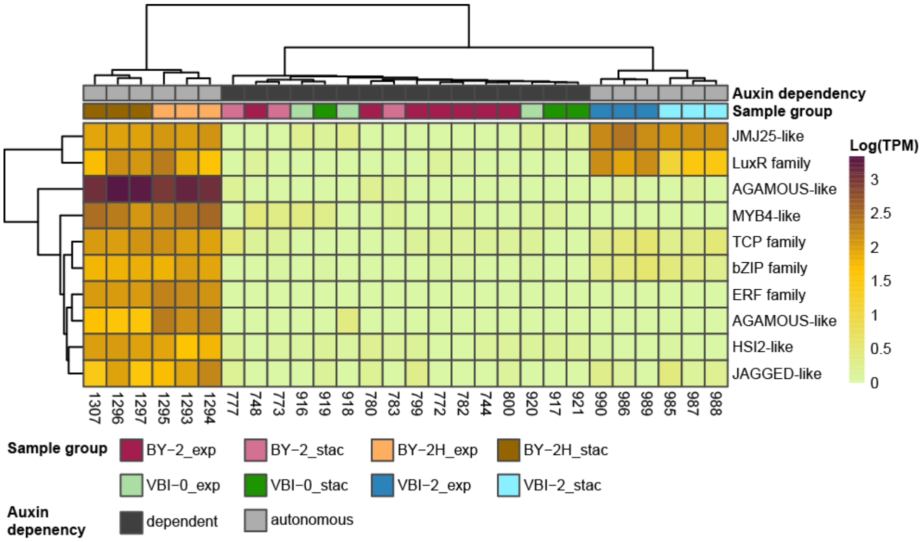

Fig. S6 Figure S6. Transcript levels (ln TPM) of top 10 most abundant transcription factors specific to VBI-2. Transcription factors in tobacco transcriptome dataset was first filtered to select only those specific to auxin habituated cell lines (TPM in all BY-2 and VBI-0 samples <= 0.5 AND TPM in all BY-2H and VBI-2 samples >= 1). Transcription of ten most abundant TFs in VBI-2_exp is shown in heatmap. Table **S6** contains list of all filtered TFs.

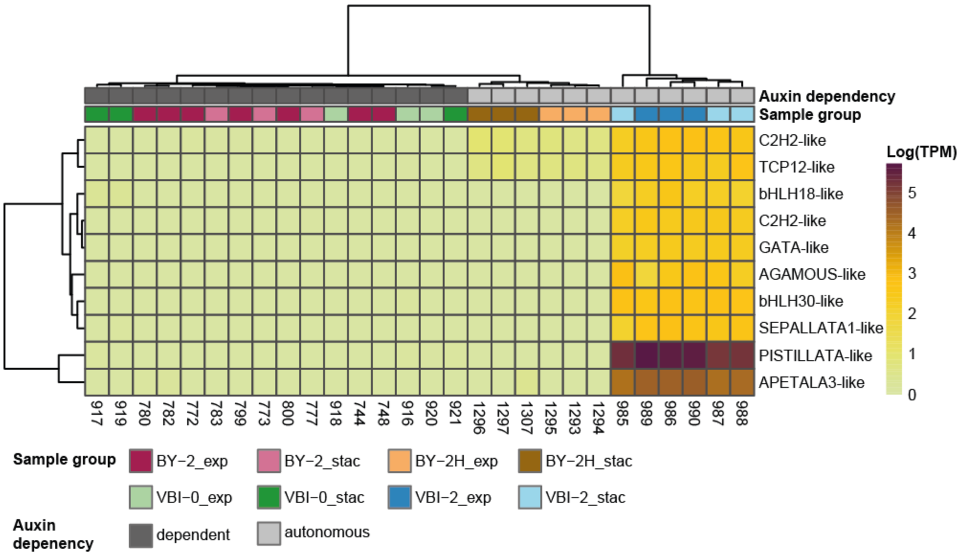

Fig. S7 Inducible overexpression of *NtTAA1* and *NtYUCCA3* allow auxin-autonomous cell proliferation in BY-2 cells (a) The relative transcription of *NtTAA1* and *NtYUCCA3* in 2-day-old *XVE*::*NtTAA1+NtYUCCA3* non-induced (DMSO) and induced (β-estradiol) cells. (b) Cell density in 2-day-old non-induced and induced *XVE*::*NtTAA1+NtYUCCA3* cells cultured in auxin-free medium. (c) Concentration of free IAA in 2-day-old non-induced and induced *XVE*::*NtTAA1+NtYUCCA3* cells cultured in auxin-free medium. (d) The representative images of 4-day-old non-induced and induced cells. Scale bar: 50 µm.

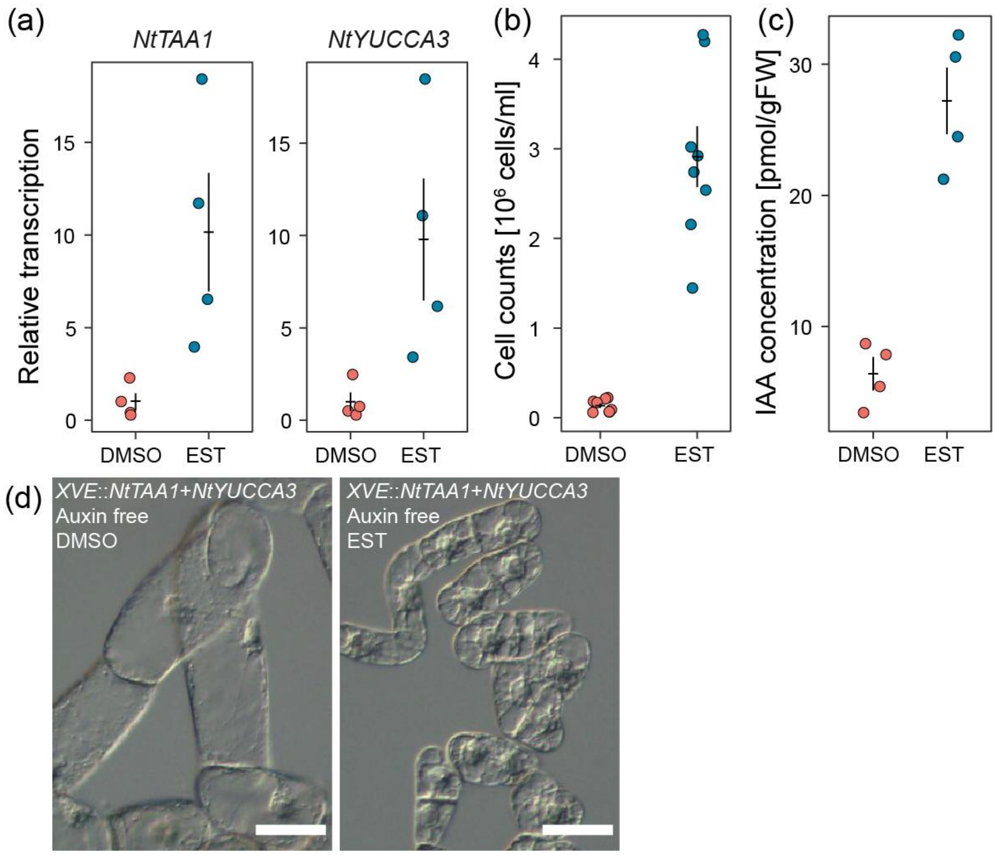

Table S1 Click here to enter text.

Methods S1 Click here to enter text.

Notes S1 Click here to enter text.

Video/Movie S1 Click here to enter text.
